# Association between low pH and unfavorable neurological outcome among out-of-hospital cardiac arrest patients treated by extracorporeal CPR: a prospective observational cohort study in Japan

**DOI:** 10.1186/s40560-020-00451-6

**Published:** 2020-05-11

**Authors:** Yohei Okada, Takeyuki Kiguchi, Taro Irisawa, Kazuhisa Yoshiya, Tomoki Yamada, Koichi Hayakawa, Kazuo Noguchi, Tetsuro Nishimura, Takuya Ishibe, Yoshiki Yagi, Masafumi Kishimoto, Hiroshi Shintani, Yasuyuki Hayashi, Taku Sogabe, Takaya Morooka, Haruko Sakamoto, Keitaro Suzuki, Fumiko Nakamura, Norihiro Nishioka, Tasuku Matsuyama, Junya Sado, Satoshi Matsui, Takeshi Shimazu, Kaoru Koike, Takashi Kawamura, Tetsuhisa Kitamura, Taku Iwami, on behalf of the CRITICAL Study Group Investigators

**Affiliations:** 1 Department of Preventive Services, School of Public Health, Kyoto University, Kyoto, Japan; 2 Department of Primary care and Emergency Medicine, Graduate School of Medicine, Kyoto University, Kyoto, Japan; 3 Kyoto University Health Service, Yoshida Honmachi, Sakyo, Kyoto, 606-8501 Japan; 4 Critical Care and Trauma Center, Osaka General Medical Center, Osaka, Japan; 5 Department of Traumatology and Acute Critical Medicine, Osaka University Graduate School of Medicine, Suita, Japan; 6 Emergency and Critical Care Medical Center, Osaka Police Hospital, Osaka, Japan; 7 Department of Emergency and Critical Care Medicine, Takii Hospital, Kansai Medical University, Moriguchi, Japan; 8 Department of Emergency Medicine, Tane General Hospital, Osaka, Japan; 9 Department of Critical Care Medicine, Osaka City University, Osaka, Japan; 10 Department of Emergency and Critical Care Medicine, Kindai University School of Medicine, Osaka, Sayama Japan; 11 Osaka Mishima Emergency Critical Care Center, Takatsuki, Japan; 12 Osaka Prefectural Nakakawachi Medical Center of Acute Medicine, Higashi-, Osaka, Japan; 13 Senshu Trauma and Critical Care Center, Osaka, Japan; 14 Senri Critical Care Medical Center, Saiseikai Senri Hospital, Suita, Japan; 15 Traumatology and Critical Care Medical Center, National Hospital Organization Osaka National Hospital, Osaka, Japan; 16 Emergency and Critical Care Medical Center, Osaka City General Hospital, Osaka, Japan; 17 Department of Pediatrics, Osaka Red Cross Hospital, Osaka, Japan; 18 Emergency and Critical Care Medical Center, Kishiwada Tokushukai Hospital, Osaka, Japan; 19 Department of Emergency and Critical Care Medicine, Kansai Medical University, Hirakata, Osaka Japan; 20 Department of Emergency Medicine, Kyoto Prefectural University of Medicine, Kyoto, Japan; 21 Division of Environmental Medicine and Population Sciences, Department of Social and Environmental Medicine, Graduate School of Medicine, Osaka University, Osaka, Japan

**Keywords:** Extracorporeal membrane oxygenation (ECMO), Percutaneous cardiopulmonary support (PCPS), Ventricular fibrillation, Extracorporeal life support, Blood gas assessment

## Abstract

**Background:**

We aimed to identify the association of pH value in blood gas assessment with neurological outcome among out-of-hospital cardiac arrest (OHCA) patients treated by extracorporeal cardiopulmonary resuscitation (ECPR).

**Methods:**

We retrospectively analyzed the database of a multicenter prospective observational study on OHCA patients in Osaka prefecture, Japan (CRITICAL study), from July 1, 2012 to December 31, 2016. We included adult OHCA patients treated by ECPR. Patients with OHCA from external causes such as trauma were excluded. We conducted logistic regression analysis to identify the odds ratio (OR) and 95% confidence interval (CI) of the pH value for 1 month favorable neurological outcome adjusted for potential confounders including sex, age, witnessed by bystander, CPR by bystander, pre-hospital initial cardiac rhythm, and cardiac rhythm on hospital arrival.

**Results:**

Among the 9822 patients in the database, 260 patients were finally included in the analysis. The three groups were Tertile 1: pH ≥ 7.030, Tertile 2: pH 6.875–7.029, and Tertile 3: pH < 6.875. The adjusted OR of Tertiles 2 and 3 compared with Tertile 1 for 1 month favorable neurological outcome were 0.26 (95% CI 0.10–0.63) and 0.24 (95% CI 0.09–0.61), respectively.

**Conclusions:**

This multi-institutional observational study showed that low pH value (< 7.03) before the implementation of ECPR was associated with 1 month unfavorable neurological outcome among OHCA patients treated with ECPR. It may be helpful to consider the candidate for ECPR.

## Background

Extracorporeal cardiopulmonary resuscitation (ECPR) is a mechanical hemodynamic support for out-of-hospital cardiac arrest (OHCA) patients using veno-arterial extracorporeal membrane oxygenation (V-A ECMO). Although this advanced resuscitation is expected to improve outcomes among patients with refractory cardiac arrest, it is invasive and expensive and requires considerable human resources [1–3]. Therefore, it is important to judge whether this is appropriate for a patient immediately after hospital arrival, based on the available information associated with neurological outcomes [4, 5].

Blood gas assessment (BGA) is performed easily and commonly to identify the treatable causes and predict prognosis in resuscitation for OHCA [6–10]. Among the factors assessed in BGA, the pH value in particular is influenced by metabolic and respiratory acidosis and is representative of hemodynamic and respiratory conditions [11]. Some observational studies show that the pH value after the return of spontaneous circulation (ROSC) is associated with neurological outcomes among OHCA patients [6–8]. These results may be helpful in considering the indication of intensive care admission or targeted temperature management after ROSC; however, the decision to start ECPR needs to be made before ROSC. Thus, these results may not be generalizable to ECPR candidates. One other observational study indicated that the pH value during resuscitation was related to neurological outcome; however, this study did not include patients with ECPR [6]. Currently, little is known about the association between pH value before the implementation of ECPR and neurological outcome among OHCA patients treated by ECPR. Our study aimed to determine the association between the pH value before implementation of ECPR and neurological outcome, among OHCA patients treated with ECPR.

## Methods

We have reported the methodology of this study according to the STrengthening the Reporting of OBservational studies in Epidemiology (STROBE) statement [12]. The Ethics Committee of Kyoto University and each participating institution approved this study protocol (R1045), and written informed consent was waived.

### Study design and settings

We performed retrospective analysis of the database of the Comprehensive Registry of Intensive Care for OHCA Survival (CRITICAL) study. This is a multicenter prospective observational study to collect pre-hospital and in-hospital data among OHCA patients in Osaka prefecture, Japan. The pre-hospital data was obtained from the All-Japan Utstein Registry of the Fire and Disaster Management Agency (FDMA) [13–16]. In-hospital data were obtained from 13 tertiary critical care medical centers (CCMCs) and 1 non-CCMC community hospital with an emergency department, all located in Osaka prefecture in Japan. Osaka prefecture is an urban area of 1905 km^2^, and it had a residential population of about 8.8 million in 2015 [17]. In Osaka prefecture, a total of 7500 OHCA cases occur every year [18], and approximately 1 in 4 OHCA patients (approximately 2000 cases or more) have been registered every year from 2012 to 2016. This registry is still ongoing, with an undefined study period. In-hospital data were recorded by the physicians in charge of the patients and were registered by the physicians or medical administrators using a predefined online form. Finally, the working group checked and confirmed the quality of data. If the data were incomplete, they were returned to each institution and completed [16]. A detailed description of the All-Japan Utstein Registry of FDMA and the CRITICAL study has been published previously [16].

### Study patients

From the CRITICAL database, we included all adult (aged ≥ 18 years) patients with OHCA due to internal medical causes, who were treated with ECPR, between July 1, 2012 and December 31, 2016. We defined ECPR as the initiation of cardiopulmonary bypass using V-A ECMO with the emergency cannulation of a large vein and artery for OHCA patients on hospital arrival during the resuscitation [19]. We excluded the following patients: those who did not receive any resuscitation or treatment in the hospital, with unavailable pre-hospital records, whose age was 17 years or less or unknown, who collapsed following cardiac arrest due to external causes such as trauma, drowning, or hanging, and those who did not undergo ECPR. We also excluded those without available BGA results before implementation of ECPR. In this cohort, the implementation of ECPR was decided by the physicians in charge of the patients or by each institution’s protocol.

### Outcome

The primary outcome of our study was 1 month survival with favorable neurological outcome, defined as Cerebral Performance Category (CPC) 1 or 2. CPC is most commonly used to evaluate neurological status as follows: category 1, good cerebral performance; category 2, moderate cerebral disability; category 3, severe cerebral disability; category 4, coma or vegetative state; and category 5, death [15].

### Data measurement and collection

From the CRITICAL database, we obtained the following clinical information: sex, age (< 65, 65–74, ≥ 75), cause of cardiac arrest (cardiac, others), witnessed by bystander (yes, no), CPR performed by bystander (yes, no), pre-hospital initial cardiac rhythm (shockable, non-shockable), cardiac rhythm on hospital arrival (shockable, non-shockable, ROSC), pH in the BGA before the implementation of ECPR, resuscitation time course, and outcomes. Age categories were defined on the basis of a government reference [17]. The pH value in venous BGA can be used interchangeably with that in arterial BGA because they are well related to each other [20, 21]. Thus, we treated them as the same. The resuscitation time courses were defined as the time from emergency call (E-call) for ambulance to hospital arrival, BGA, and start of ECPR in the hospital. The included patients were divided into three groups of approximately equal size, based on the pH value in the BGA (Tertiles 1 to 3).

### Potential bias

We excluded patients who lacked BGA data from the main analysis (complete case analysis). If data are missing completely at random, excluding patients with missing data does not lead to biased results; thus, it may be acceptable [22]. However, if the missing does not occur at random and depends on the outcome and exposure, then it would introduce selection bias [22]. Therefore, to demonstrate the robustness of our results and compensate for the risk of selection bias, we described the characteristics of patients with missing data and performed a sensitivity analysis presuming that the missing of data depended on exposure and outcome; this has been described in Additional File 1 (Details of the sensitivity analysis are also described in Additional File 1).

### Statistical analysis

We described the patients’ characteristics in each patient group. To identify the associations of the pH with the primary outcome, we calculated crude odds ratios (OR) and adjusted OR with 95% confidence intervals (CI) of each patient group for the outcome, using a logistic regression model. We adjusted for the following potential confounders: sex (male, female), age (< 65, 65–74, and ≥ 75), witnessed by bystander, CPR by bystander, pre-hospital initial cardiac rhythm (shockable, non-shockable), and cardiac rhythm on hospital arrival (shockable, non-shockable, and ROSC). Moreover, for better understanding of the results, we also calculated the area under the curve of the receiver operating characteristic curve (AUC_ROC) to predict the neurological outcome, treating the pH values as continuous variables. We also described the characteristics of those who had favorable neurological outcomes in each group.

We did not estimate a sample size because our analysis involved secondary usage of already available data [12]. All statistical results were considered significant at a two-sided *P* value of < 0.05. All statistical analyses were performed using the JMP Pro® 14 software (SAS Institute Inc., Cary, NC, USA).

## Results

### Study participants

**Fig. 1:**
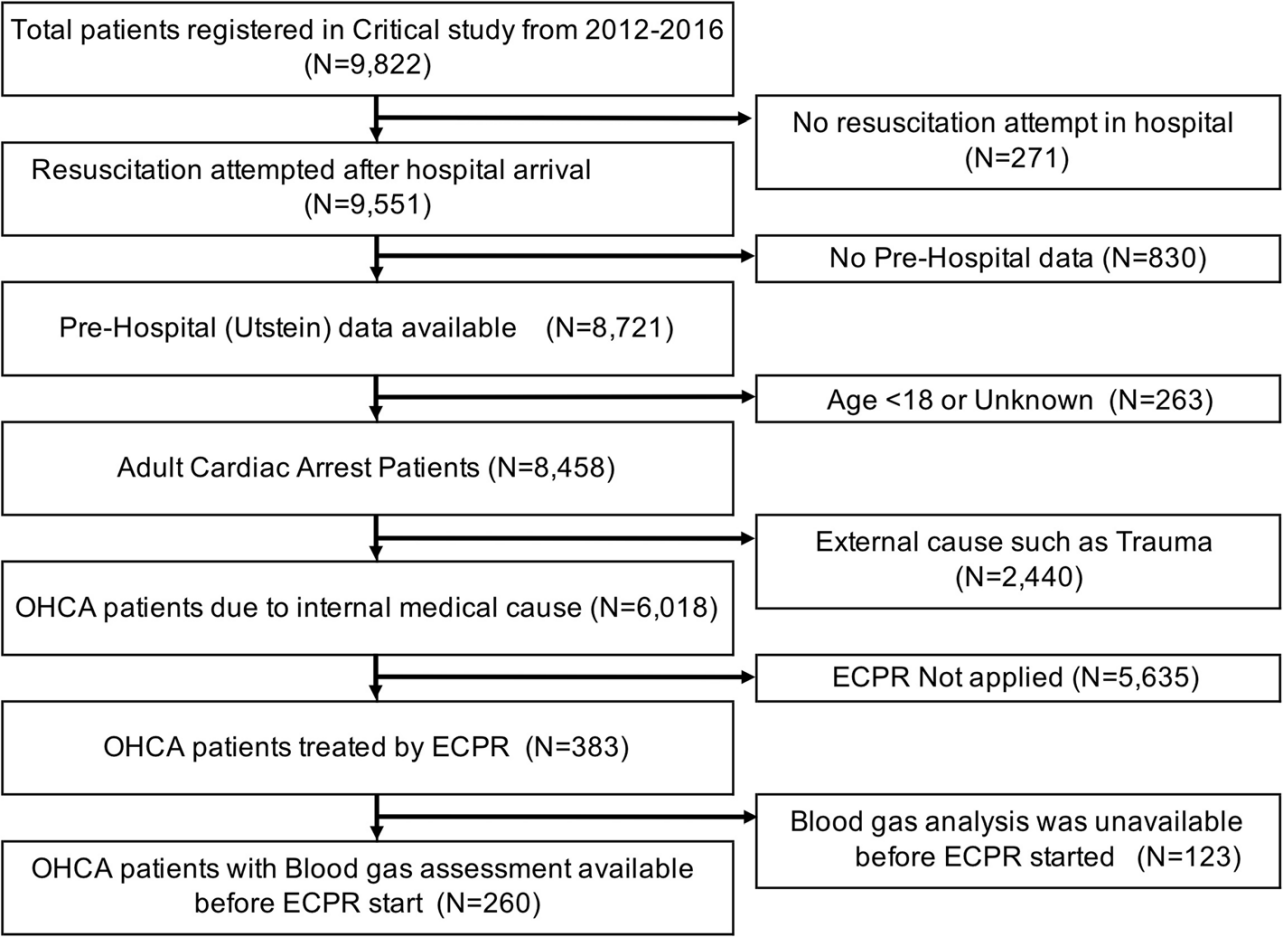
Study flow chart

Among the 9822 patients in the CRITICAL database, 260 were finally included in the analysis (Fig. 1). The three groups into which the included patients were divided were Tertile 1: pH ≥ 7.03, Tertile 2: pH 6.875–7.029, and Tertile 3: pH < .875. The characteristics of the patients are shown in Table 1. In summary, the patients in Tertile 3 (pH < 6.875) were relatively young (age, median, [IQR] 55.5, [46–66] years), compared with those in Tertile 1 (pH ≥ 7.03) (67 [56.8–75.3] years) and Tertile 2 (pH 6.875–7.029) (63.5 [49.0–69.8] years). The other parameters were substantially similar among groups.

**Table 1:** The clinical characteristics

	Total	pH on blood gas analysis before ECPR started		
		Tertile 1	Tertile 2	Tertile 3
Parameters		(≥ 7.03)	(7.029–6.875)	(< 6.875)
	(***N*** = 260)	(***N*** = 86)	(***N*** = 88)	(***N*** = 86)
**Men**	197 (75.8%)	63 (73.3%)	65 (73.9%)	69 (80.2%)
**Age, years**	62.5 (49–71)	67 (56.8-75.3)	63.5 (49–69.8)	55.5 (46–66)
18–65	146 (56.2%)	38 (44.2%)	48 (54.5%)	60 (69.8%)
65–74	72 (27.7%)	22 (25.6%)	30 (34.1%)	20 (23.3%)
≥ 75	42 (16.2%)	26 (30.2%)	10 (11.4%)	6 (7.0%)
**Cause of cardiac arrest**				
Cardiac	245 (94.2%)	81 (94.2%)	83 (94.3%)	81 (94.2%)
**Pre-hospital information**				
Bystander witness	206 (79.2%)	66 (76.7%)	71 (80.7%)	69 (80.2%)
Bystander CPR	120 (46.2%)	36 (41.9%)	42 (47.7%)	42 (48.8%)
Shockable on initial rhythm	175 (67.3%)	58 (67.4%)	63 (71.6%)	54 (62.8%)
Advanced airway	110 (69.6%)	33 (67.3%)	32 (69.6%)	45 (71.4%)
**In-hospital information**				
**Cardiac rhythm on arrival**				
ROSC	22 (8.5%)	14 (16.3%)	6 (6.8%)	2 (2.3%)
Shockable	121 (46.5%)	39 (45.3%)	41 (46.6%)	41 (47.7%)
Non-shockable	117 (45%)	33 (38.4%)	41 (46.6%)	43 (50%)
**pH value before ECPR start**	6.95 (6.83–7.08)	7.13 (7.08–7.20)	6.95 (6.91–6.99)	6.78 (6.72–6.83)
**Time course, min**				
E-call to hospital arrival	31 (25–38)	30 (22–36)	30 (26–37.5)	35 (28–44)
E-call to collect BGA	39 (32–48)	35 (29–45)	39.5 (33.3–46.0)	43.5 (36.0–54.0)
E-call to start ECPR	60 (51–79)	60 (50.8–90.5)	61.5 (51.3–82)	59 (51.0–71.8)

Continuous value is described as median and IQR. Categorical variables are number and percentage. No missing value in these parameters

*IQR* interquartile range, *CPR* cardio-pulmonary resuscitation, *ROSC* return of spontaneous circulation, *PEA* pulseless electrical activity, *BGA* blood gas analysis, *E-call* call to the emergency service, *ECPR* extra-corporeal circulatory support during the CPR

### Primary outcome

The primary outcome (1 month survival with favorable neurological outcome) was 27.9% (24/86) in Tertile 1 (pH ≥ 7.03), 10.2% (9/88) in Tertile 2 (pH 6.875 to 7.029), and 9.3% (8/86) in Tertile 3 (pH < 6.875).

**Fig. 2:**
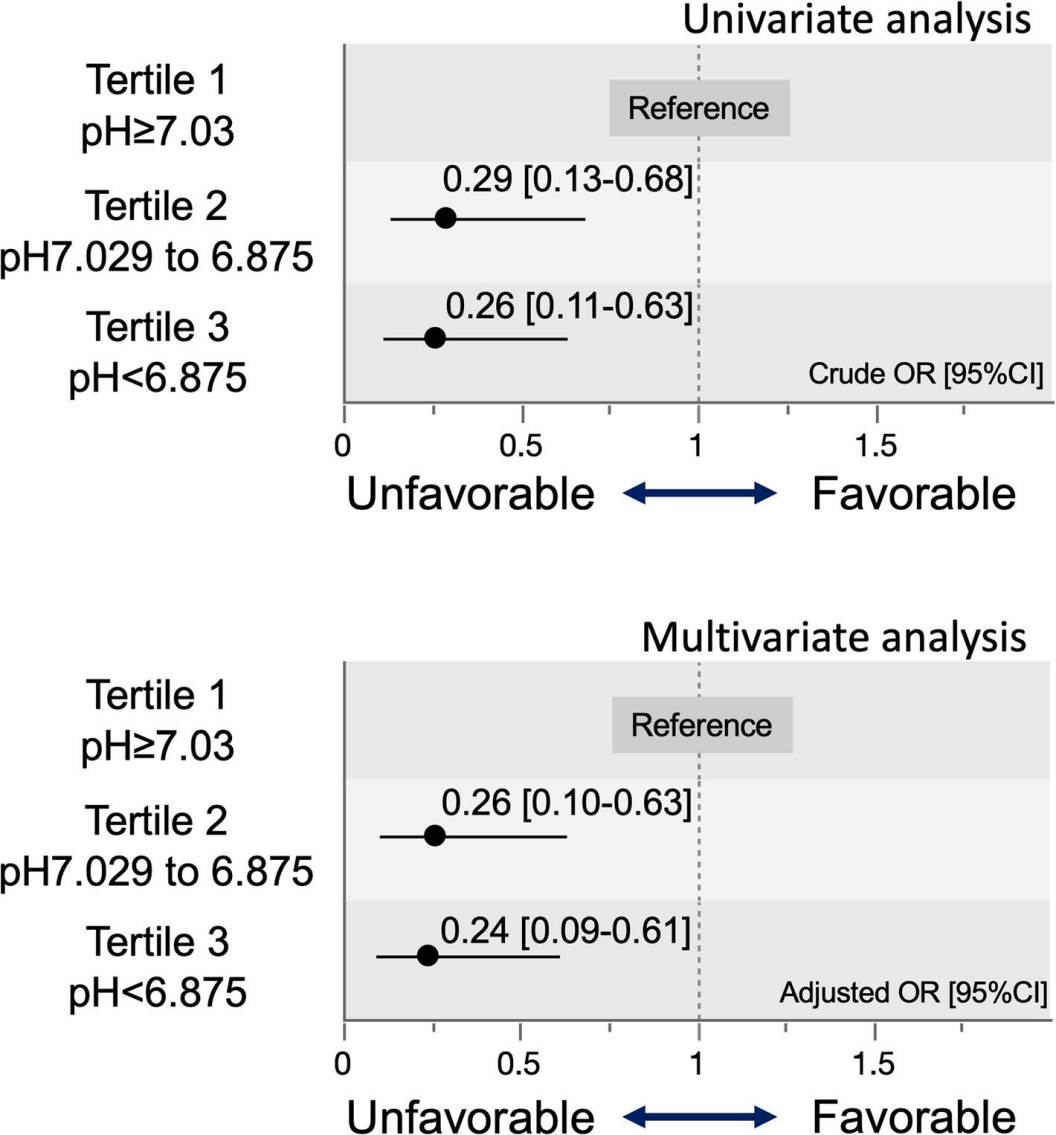
Crude and adjusted odds ratios and 95% CI of Tertiles 2 and 3 for the primary outcome. Adjusted by sex, age, witness of collapse, bystander CPR, prehospital initial rhythm, and initial rhythm on hospital arrival. OR, odds ratio; CI, confidence interval

The crude OR with 95% CI for primary outcome of Tertiles 2 and 3, compared with Tertile 1 for reference, were 0.29 (95% CI 0.13–0.68) and 0.26 (95% CI 0.11–0.63), respectively (Fig. 2). Adjusted ORs with 95% CI for primary outcome of Tertiles 2 and 3 compared with Tertile 1 were 0.26 (95% CI 0.10–0.63) and 0.24 (95% CI 0.09–0.61), respectively (Fig. 2). According to these results, Tertile 2 (pH 6.875–7.029) and Tertile 3 (pH < 6.875) were associated with unfavorable neurological outcome, compared with Tertile 1 (pH ≥ 7.03). The crude and adjusted ORs of the other covariates are provided in the supplementary file. The discrimination ability of pH (AUC_ROC) was 0.675 [95% CI 0.573–0.763].

### The characteristics of patients with favorable neurological outcome

The characteristics of patients with favorable neurological outcomes are shown in Table 2. Those in Tertiles 2 and 3 were more likely to be young, have OHCA witnessed by bystanders, and have ECPR implemented early after arrival.

**Table 2:** The characteristics in the patients with neurological favourable outcome

	pH on blood gas analysis before ECPR started		
Parameters	Tertile 1	Tertile 2	Tertile 3
	(≥ 7.03)	(7.029–6.875)	(< 6.875)
	(***N*** = 24)	(***N*** = 9)	(***N*** = 8)
**Men**	19 (79.2%)	5 (55.6%)	7 (87.5%)
**Age, years**	62.5 (51.5–68.8)	58.0 (51.5–69.5)	50.5 (33.0–60.0)
18-65 years	13 (54.2%)	6 (66.7%)	7 (87.5%)
65–74	8 (33.3%)	3 (33.3%)	1 (12.5%)
≥ 75	3 (12.5%)	0 (0.0%)	0 (0.0%)
**Cause of cardiac arrest**			
Cardiac	21 (87.5%)	7 (77.8%)	8 (100%)
**Pre-hospital information**			
Bystander witness	14 (58.3%)	9 (100%)	7 (87.5%)
Bystander CPR	10 (41.7%)	5 (55.6%)	4 (50%)
Shockable on initial rhythm	18 (75%)	6 (66.7%)	7 (87.5%)
Advanced airway	6 (54.5%)	0 (0.0%)	4 (80.0%)
**In-hospital information**			
**Cardiac rhythm on arrival**			
ROSC	6 (25%)	2 (22.2%)	0 (0%)
Shockable	15 (62.5%)	4 (44.4%)	6 (75%)
Non-shockable	3 (12.5%)	3 (33.3%)	2 (25%)
**pH value before ECPR start**	7.14 (7.10–7.26)	6.96 (6.93–7.00)	6.75 (6.71–6.83)
**Time course, min**			
E-call to hospital arrival	29.5 (19.3–35.0)	27.5 (19.5–38.8)	25.5 (19.3–33.0)
E-call to collect BGA	33 (26.8–41.3)	43 (31–51)	36 (32.3–45.8)
E-call to start ECPR	63 (51–191.5)	48 (45.5–65.0)	49 (46.3–58.5)

Continuous value is described as median and IQR. Categorical variables are number and percentage

*IQR* interquartile range, *CPR* cardio-pulmonary resuscitation, *ROSC* return of spontaneous circulation, *PEA* pulseless electrical activity, *BGA* blood gas analysis, *E-call* call to the emergency service, *ECPR* extra-corporeal circulatory support during the CPR

### Sensitivity analysis

Under this assumption, Tertile 2 (pH 6.875–7.029) and Tertile 3 (pH < 6.875) were also independently associated with neurological outcome (Additional file 1). This result demonstrates the robustness of this association, despite the exclusion of the patients with missing BGA.

## Discussion

### Key observations

This multi-institutional observational study including 14 emergency departments showed that the pH value before the implementation of ECPR was associated with 1 month neurological outcomes among OHCA patients treated with ECPR. It may be helpful to consider the candidate for ECPR.

### Interpretation of the results

We suggest that our results may be explained as follows: severe acidemia, including metabolic and respiratory acidosis, is representative of the severe conditions of hypoperfusion of vital organs and insufficient discharge of carbon dioxide during resuscitation, and these conditions may lead to cerebral injury or multiple organ failure and unfavorable outcomes. Metabolic acidosis, particularly lactic acidosis, is caused by inadequate oxygen delivery, impaired tissue oxygenation, and anaerobic glycolysis [23]. In cardiac arrest patients, it may be affected by low cardiac output by chest compression during resuscitation [24]. Some observational studies have reported that metabolic acidosis after ROSC is correlated with the duration from arrest to ROSC and is associated with neurological outcome among OHCA patients [8, 25–28]. Respiratory acidosis, the other cause of severe acidemia, indicates inadequate discharge of carbon dioxide and is mostly caused by low venous return by chest compression and insufficient alveolar ventilation during resuscitation [24, 29]. Previous observational studies also reported that respiratory acidosis is associated with cerebral injury and unfavorable neurological outcomes among post-cardiac arrest patients or those with head trauma injury [30, 31]. Thus, it is possible that lower pH values may represent a longer duration of cardiac arrest, lower cerebral blood flow and venous return, and insufficient ventilation; these conditions are associated with an unfavorable neurological outcome.

### Clinical implication

We conclude that pH measurement may be helpful to judge the indication of ECPR. If OHCA patients have a pH value higher than 7.03, they have a higher probability of favorable neurological outcome. The results of BGA are objective, reproductive, and available as soon as a blood sample is collected. Further, when ECPR is attempted, obtaining access to the femoral artery enables continual collection of blood samples. Therefore, pH measurement can be easily applied to real clinical settings.

It should be noted that in our results, some patients with severe acidemia did survive with favorable neurological outcomes. These patients were relatively young, with shockable rhythms and OHCA witnessed by bystanders. A previous case series also reported that some patients with severe acidemia could achieve good recovery from OHCA in some situations [32]. Furthermore, in our study, the discrimination ability of pH (AUC_ROC) was 0.675 [95% CI 0.573–0.763], which is not adequately high for predicting the neurological outcome definitely. According to these findings, physicians considering the indication of ECPR should not make quick decisions based only on the pH value.

### Strengths and limitations

Compared with previous studies, the strength of our study was that we could identify the association between pH values and neurological outcomes by adjusting for potential confounders. Among ECPR patients, a previous systematic review reported that witnessed cardiac arrest, CPR performed by bystander, initial shockable rhythm, arrest to ECPR duration, and higher pH value during resuscitation may be potential predictors for survival [33]. In the meta-analysis including five observational studies in this review, there were statistical differences between survivors and non-survivors based on pH value (7.16 ± 0.04 vs 7.01 ± 0.06, mean difference 0.14 [95% CI 0.08–0.21]) during resuscitation [33]. However, this analysis did not consider the effect of confounding. Further, it did not identify an association with neurological outcome. Conversely, our analysis adjusted for several major confounders using a logistic regression model and showed the association with neurological outcome. Therefore, our results showed a more robust association than previous studies.

Our study also has several limitations. First, the timing of collecting blood samples and the collecting sites (arterial or venous) were not strictly defined; this may have caused measurement bias. Second, our sample size and the number of events were limited. For more precise estimation, a larger sample size would be better. Third, some potential unmeasured confounders may influence the results. Fourth, the indication of ECPR was decided by each physician or according to each institution’s protocol. Thus, there may be selection bias. Finally, this registry was derived from a critical care center in Osaka, Japan; it is unclear as to what extent the results can be generalized to other populations or other settings.

## Conclusions

Our study showed that a lower pH value (< 7.03) was associated with unfavorable neurological outcomes among OHCA patients treated by ECPR. Our results may be helpful in deciding the indication of ECPR.

## Supplementary information

**Additional file 1: S-Table 1.** The characteristics of patients with missing BGA. **S-Table 2.** Original cohort (*N*=260). **S-Table 3.** Expected outcome among the excluded patients in the assumption (*N*=123). **S-Table 4.** The crude and adjusted ORs of other covariates. **S-Table 5.** The results of sensitivity analysis. S-Figure. Flow chart in sensitivity analysis.

## Data Availability

Not applicable

## Abbreviations

BGA: Blood gas assessment
CCMC: Critical care medical center
CPC: Cerebral Performance Category
CRITICAL: Comprehensive Registry of Intensive Care for OHCA Survival
ECPR: Extracorporeal cardiopulmonary resuscitation
FDMA: Fire and Disaster Management Agency
OHCA: Out-of-hospital cardiac arrest
ROSC: Return of spontaneous circulation
STROBE: STrengthening the Reporting of OBservational studies in Epidemiology
V-A ECMO: veno-arterial extracorporeal membrane
