# Supplementary material for: Association between low pH and unfavorable neurological outcome among out-of-hospital cardiac arrest patients treated by extracorporeal CPR: a prospective observational cohort study in Japan
Source: J Intensive Care. 2020 May 11;8:34. doi: 10.1186/s40560-020-00451-6 (PMC7212572; doi:10.1186/s40560-020-00451-6)
Supplement: Supplementary file 1 — Additional file 1: S-Table 1. The characteristics of patients with missing BGA. S-Table 2. Original cohort (N=260). S-Table 3. Expected outcome among the excluded patients in the assumption (N=123). S-Table 4. The crude and adjusted ORs of other covariates. S-Table 5. The results of sensitivity analysis. S-Figure. Flow chart in sensitivity analysis. [file 40560_2020_451_MOESM1_ESM.docx]

**[Additional file1]**

**Supplementary Methods: Assessment for potential selection bias**

We excluded patients who lacked blood gas assessment (BGA) data from the main analysis (complete case analysis). If data are missing completely at random (MCAR) or at random (MAR), excluding patients with missing data does not lead to biased results; thus, it can be acceptable.[1] However, if the missing happens not at random (MNAR), and depends on the exposure and outcome, then it would introduce selection bias.[1] Therefore, in order to demonstrate the robustness of our results and compensate for the risk of selection bias due to the exclusion, we describe the characteristics of patients with missing data (S-Table1) Moreover, we performed a probabilistic bias analysis as sensitivity analysis, using the scenario that missing depended on exposure and outcome.

**S-Table 1. The characteristics of patients with missing BGA**

|  | **Parameters** | **Total (N=123)** |
| --- | --- | --- |
| **Men** | | 99 (80.5%) |
| **Age, y** | | 58 (46-68) |
|  | 18-65 y.o | 78 (63.4%) |
|  | 65-74 | 31 (25.2%) |
|  | ≥ 75 | 14 (11.4%) |
| **Cause of cardiac arrest** | |  |
|  | Cardiac | 111 (90.2%) |
| **Pre-hospital information** | |  |
|  | Bystander witness | 96 (78.0%) |
|  | Bystander CPR | 40 (32.5%) |
|  | Shockable on initial rhythm | 93 (75.6%) |
|  | Advanced airway | 57 (46.3%) |
| **In-hospital information** | |  |
| **Cardiac rhythm on arrival** | |  |
|  | ROSC | 6 (4.9%) |
|  | Shockable | 65 (52.8%) |
|  | Non-shockable | 52 (42.3%) |
| **Time course, min** | |  |
|  | E-call to Hospital arrival | 30.0 (24.0-38.0) |
|  | E-call to start ECPR | 51.0 (43.0-66.5) |
| **One-month favourable neurological outcome** | | 28 (22.8%) |

Continuous variables are given as median and IQR. Categorical variables are given as number and percentage.

IQR: interquartile range, CPR: cardiopulmonary resuscitation, ROSC: return of spontaneous circulation, BGA: blood gas analysis, E-Call: call to the emergency service, ECPR: extra-corporeal circulatory support during CPR

**Sensitivity analysis**

Assumption: Missing depends on the exposure and outcome.

We assumed that the patients whose pH values were low, but whose outcomes were favourable, or the patients whose pH values were high, but whose outcomes were unfavourable, tended to have missing BGA. In such situations, there would be weaker or no association between exposure and outcome among the patients with missing BGA data. Then, excluding the patients with missing data would lead to selection bias. [2]

We assumed the most conservative scenario, that there was no association between the pH value and outcome among the patients with missing data, which means that odd ratios (OR) of Tertiles 2 and 3 for the outcome (using Tertile 1 as reference) are 1.0 and 1.0, respectively (S-Table 3). Concretely, we assigned the excluded patients into Tertiles 1~3 randomly and equally (1:1:1) regardless of their outcome (S-Table 3).

Then, we added those to the original cohort, and performed multivariate logistic regression analysis to calculate the adjusted OR of Tertiles 2 and 3 with reference to Tertile 1 for favourable neurological outcome, the same as the primary analysis (S-Figure). We repeated this 1000 times to estimate the adjusted OR and 95% CI.

**S-Table 2. Original cohort (N=260)**

|  | Favourable outcome + | Favourable outcome - | OR |
| --- | --- | --- | --- |
| Tertile 1 | 24 | 62 | ref |
| Tertile 2 | 9 | 79 | 0.29 |
| Tertile 3 | 8 | 78 | 0.26 |
| sum | 41 | 219 | 260 |

**S-Table 3 Expected outcome among the excluded patients in the assumption (N=123)**

|  | Expected Favourable outcome + | Expected Favourable outcome - | OR |
| --- | --- | --- | --- |
| Tertile 1 | 9.3 | 31.7 | ref |
| Tertile 2 | 9.3 | 31.7 | 1.0 |
| Tertile 3 | 9.3 | 31.7 | 1.0 |
| sum | 28 | 95 | 123 |

OR: odds ratio, OR for favourable outcome + with reference to Tertile 1.

There is no association between Tertile 1~3 and outcome among the patients with missing data (N=123).

Patients with missing data were assigned to Tertiles 1~3 equally and randomly. (S-Table 3)

**S-Figure. Flow chart in sensitivity analysis**


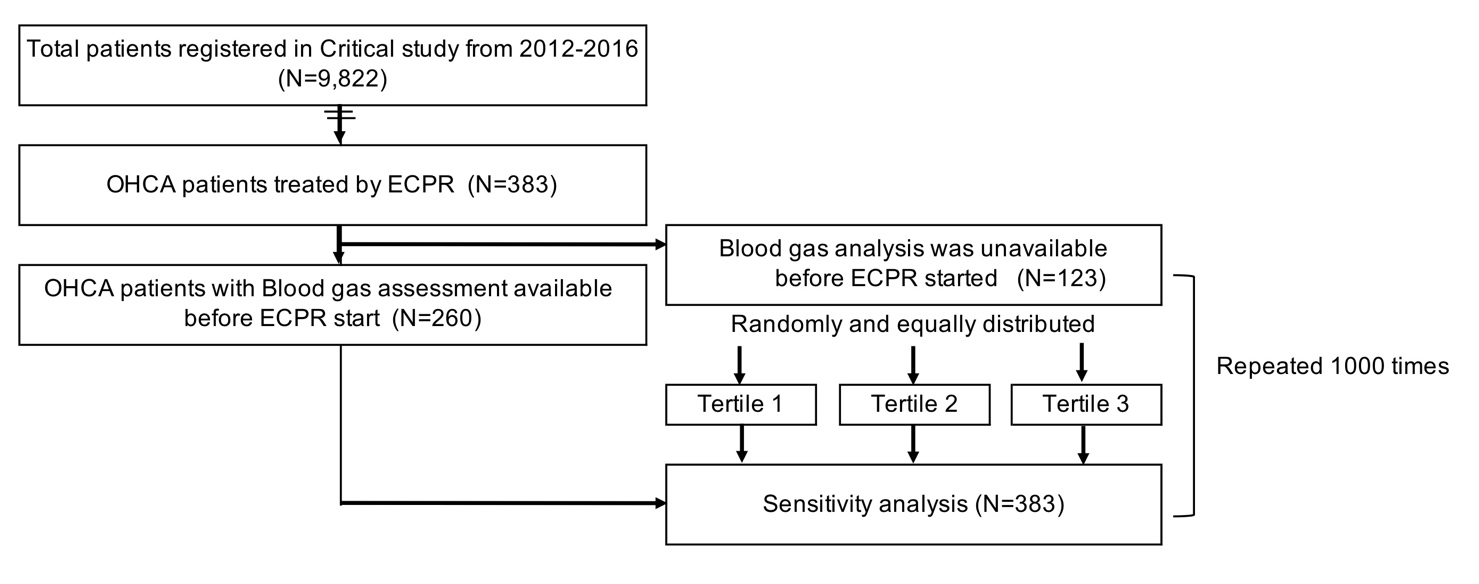


**Supplementary Results**

**S-Table 4. The crude and adjusted ORs of other covariates**

| Variables | | Crude OR | 95%CI | | Adjusted OR | 95%CI | |
| --- | --- | --- | --- | --- | --- | --- | --- |
| Men/ Women | | 0.99 | 0.46 | 2.15 | 1.03 | 0.38 | 2.77 |
| Age | |  |  |  |  |  |  |
|  | 18-59 | Ref |  |  | Ref |  |  |
|  | 60-79 | 0.74 | 0.37 | 1.45 | 0.28 | 0.11 | 0.67 |
|  | ≥ 80 | 0.27 | 0.03 | 2.16 | 0.15 | 0.02 | 1.37 |
| Bystander witness (Yes/No) | | 0.67 | 0.31 | 1.43 | 0.66 | 0.27 | 1.62 |
| Bystander CPR (Yes/No) | | 1.01 | 0.52 | 1.97 | 0.80 | 0.36 | 1.78 |
| Cardiac rhythm on arrival | |  |  |  |  |  |  |
|  | ROSC | Ref |  |  | Ref |  |  |
|  | Shockable | 7.79 | 2.52 | 24.03 | 8.51 | 2.21 | 32.74 |
|  | PEA/Asystole | 3.55 | 1.53 | 8.24 | 5.05 | 1.80 | 14.16 |
| pH in BGA before ECPR | |  |  |  |  |  |  |
|  | Tertile 1 (≥7.030) | Ref |  |  | Ref |  |  |
|  | Tertile 2 (6.875-7.029) | 0.29 | 0.13 | 0.68 | 0.22 | 0.08 | 0.57 |
|  | Tertile 3 (<6.875) | 0.26 | 0.11 | 0.63 | 0.17 | 0.06 | 0.51 |
| Time from call to hospital arrival /min | | 0.99 | 0.97 | 1.01 | 0.98 | 0.96 | 1.01 |

Ref: reference, CPR: cardiopulmonary resuscitation, ROSC: return of spontaneous circulation, PEA: Pulseless electrical activity, BGA: blood gas analysis, Call: call to the emergency service, ECPR: extra-corporeal circulatory support during CPR

**Results of sensitivity analysis**

Under this assumption and 1,000 times repeated simulation, mean crude and adjusted OR with 95% CI for primary outcome of Tertiles 2 and 3 compared with Tertile 1 were calculated as below (S-Table 4). According to these results, the Tertile 2 (pH 6.875-7.029) and Tertile 3 (pH<6.875) were also independently associated with unfavourable neurological outcome. This result demonstrates the robustness of this association, despite the exclusion of the patients with missing BGA.

**S-Table 5. The results of sensitivity analysis**

| Variables | Mean Crude OR | 95% CI | Mean Adjusted OR | 95% CI |
| --- | --- | --- | --- | --- |
| Tertile 1 | reference |  | reference |  |
| Tertile 2 | 0.46 | 0.31-0.70 | 0.46 | 0.29-0.69 |
| Tertile 3 | 0.45 | 0.29-0.66 | 0.46 | 0.29-0.70 |

Adjusted by sex, age, witness of collapse, bystander CPR, prehospital initial rhythm, and initial rhythm on hospital arrival

Assumption (Missing not at random): Assignment of the excluded patients into Tertiles 1~3 randomly and equally.

Reference

[1] Hughes RA, Heron J, Sterne JAC, Tilling K. Accounting for missing data in statistical analyses: multiple imputation is not always the answer. International Journal of Epidemiology 2019;48(4):1294-304.

[2] Lash TL. Applying quantitative bias analysis to epidemiologic data*.* S.l.: SpringerLink ebooks - Mathematics and Statistics; 2009.
